# Ceftriaxone Use Evaluation in Western Zone Tigray Hospitals, Ethiopia: A Retrospective Cross-Sectional Study

**DOI:** 10.1155/2023/7688896

**Published:** 2023-11-13

**Authors:** Tewodros Shegute, Mulugeta Hiruy, Haftom Hadush, Leake Gebremeskel

**Affiliations:** ^1^Pharmacology and Toxicology Unit, Department of Pharmacy, College of Health Sciences, Aksum University, Aksum, Ethiopia; ^2^Department of Biomedical Sciences, College of Health Sciences, Aksum University, Aksum, Ethiopia; ^3^Department of Medical Laboratory Technology, College of Health Sciences, Aksum University, Aksum, Ethiopia

## Abstract

**Background:**

Drug use evaluation is a systematic approach to determining the appropriateness of drug use, identifying drug therapy problems, and proposing interventions. Ceftriaxone is one of the most widely used drugs in hospitals, requiring the performance of drug use evaluation. This study was aimed at evaluating the appropriateness of ceftriaxone use at Kahsay Abera and Mearg hospitals in the Western zone of Tigray, Ethiopia.

**Methods:**

An institution-based retrospective cross-sectional study design was conducted in both hospitals from December 2015 to August 2016 using standardized and pretested data collection formats. Systematic random sampling was used, and a total of 800 patients' medication records (patients who took ceftriaxone) from both hospitals (400 each) were assessed in this study. Statistical analysis was performed by using the statistical package for social sciences (version 20).

**Results:**

The overall appropriateness of ceftriaxone use in Kahsay Abera and Mearg hospitals was 247 (61.75%) and 252 (63.0%), respectively. The majority of inappropriate use of ceftriaxone was noted in indication errors at both Kahsay Abera (71.2%) and Mearg hospitals (52.0%). The treatment duration in Kahsay Abera (69%) and Mearg hospitals (88%) was in the range of 2-7 days. Mostly, a 2-gram ceftriaxone daily dose was prescribed in both Kahsay Abera and Mearg hospitals, accounting for 285 (71.25%) and 318 (79.5%), respectively. In this study, the top three diseases, indicated for ceftriaxone in both hospitals, were typhoid fever, urinary tract infection, and pneumonia in descending order. Among the medications coadministered with ceftriaxone, the top three coprescribed drugs with ceftriaxone in Kahsay Abera Hospital were metronidazole (17.25%), tramadol 68 (11.28%), and diclofenac (8.96%), but in Mearg Hospital, next to metronidazole, drugs like paracetamol and doxycycline were the most common coprescribed medicines along with ceftriaxone.

**Conclusion:**

The appropriateness of ceftriaxone use in Kahsay Abera and Mearg hospitals was 247 (61.8%) and 252 (63%), respectively, in which about one-third of patients' charts were not compliant with the standard treatment guidelines of Ethiopia for general hospitals. In Kahsay Abera and Mearg hospitals, the empiric use of ceftriaxone was 262 (65.5%) and 375 (93.8%), respectively.

## 1. Background

Irrational use of antimicrobials causes serious problems such as microorganism resistance to antimicrobials and medication therapy problems which may lead to several side effects contributing to increased morbidity and mortality in addition to economic burden (increment in drug cost) [1, 2]. Knowing the relationship between emerging bacterial resistance and antimicrobial use requires monitoring antibiotics both locally and nationally [3]. Drug utilization review studies are suitable means of determining the appropriateness of drug use, identifying drug therapy problems, and proposing proper solutions [1]. Drug use evaluation (DUE) is an ongoing, systematic, criteria-based program of medicine evaluations that helps ensure appropriate medicine use [4, 5]. The DUE process involves the use of qualitative and quantitative terms to describe users of a given drug or class of drugs [6]. The misuse of 3^rd^ generation cephalosporins, particularly ceftriaxone, has led to treatment failures in certain bacterial infections [7, 8].

Ceftriaxone is a semisynthetic third-generation cephalosporin which has a bactericidal effect by inhibiting bacterial cell wall synthesis [9]. It is one of the 3^rd^ generation of cephalosporins that is mostly ordered due to its low toxicity and high efficacy against a wide range of bacterial infections [10]. Ceftriaxone is clinically used in Ethiopia in the management of serious infections due to sensitive bacteria, including septicemia, pneumonia, meningitis, surgical prophylaxis, prophylaxis of meningococcal meningitis, and gonorrhea [11]. Resistance of bacteria to ceftriaxone occurs through the production of beta-lactamase enzymes to hydrolyze the beta-lactam ring that is essential for its action [12, 13].

Infectious diseases are threats to all societies, irrespective of age, gender, ethnicity, education, and socioeconomic status. They are the most common causes of morbidity and mortality in developing countries, and their treatment also imposes a huge financial burden on societies [5, 14, 15]. With the increasing quantity and variety of pharmaceuticals available today in both developed and developing countries, their potential for inappropriate use is a growing concern [16–18]. Misuse of antimicrobial agents has numerous detrimental effects on the patient, society, and healthcare system since they are widely used [19]. Managing the use of antibiotics helps reduce antimicrobial resistance and other drug use problems [20]. The DUE helps ensure correct prescribing and use of drugs by detecting inappropriate use of medications and proposing corrective measures [21]. Resistant infections have a high impact in low-income countries including Ethiopia since they cannot afford more recent and expensive antibiotics [8]. The aim of this study was to evaluate the appropriateness of ceftriaxone utilization in Kahsay Abera and Mearg hospitals with reference to the standard treatment guideline (STG) of Ethiopia for general hospitals. As this study is new in both Kahsay Abera and Mearg hospitals, it will help in identifying improper aspects of ceftriaxone use, and then it could serve as a baseline for further antibiotic stewardship studies.

## 2. Methods

### 2.1. Study Setting

This study was conducted in Kahsay Abera and Mearg hospitals in the Western zone of Tigray, Ethiopia. Kahsay Abera Hospital is situated in Setit Humera town, 1370 km far from Addis Ababa, the capital city of Ethiopia, and 580 km from Mekelle, the capital city of the Tigray region. It has 210 beds and provides several medical services for an estimated 742,000 catchment population including migrants from Sudan and Eritrea. Mearg Hospital is located in Tsegede woreda, Dansha town, 1459 km away from Addis Ababa. It serves an estimated 299,594 catchment population including migrants; it has 134 beds in all wards.

### 2.2. Study Design and Period

A retrospective cross-sectional study design was employed to assess the ceftriaxone use in patients who were treated in Kahsay Abera and Mearg hospitals from December 2015 to August 2016.

### 2.3. Inclusion and Exclusion Criteria

In this study, all patients who had received at least one course of any dose of ceftriaxone treatment within twelve months prior to the data collection were considered. Records of incomplete information in the main variables and patients whose clinical card could not be located from the record office were excluded.

### 2.4. Sample Size Determination and Sampling Procedure

The sample size was determined using a single population proportion formula with a proportion (*P* = 0.5) of inappropriate ceftriaxone utilization and a confidence level of 95% with a *z*-value of 1.96 (*P* = 0.05) with 10% contingency, and systematic random sampling was employed. The first patients' medical chart was selected using a simple random sampling technique, then every 3^rd^ chart was selected until the desired sample size was reached; 23 medical cards (in each hospital) were incomplete and were excluded; hence, only 400 cards were analyzed in each hospital. The study population was the medication records of all patients taking ceftriaxone at Kahsay Abera and Mearg hospitals during the study period, and patients who got at least one dose of ceftriaxone were included in the study.

### 2.5. Data Collection Form

The data collection format was developed based on World Health Organization (WHO) criteria [22] for drug use evaluation and the manual for drug and therapeutics committee training course by Management Science for Health (MSH) [23]. The STG of 2014 for general hospitals was used to validate the tool for ceftriaxone in terms of indication, dosage, frequency, and duration of treatment [19, 24].

### 2.6. Data Collection Procedure

Before beginning data collection, the card number, age, gender, and treatment date records were obtained from patient registration documents. Then, patients treated with ceftriaxone were traced back to the records office of the hospitals using the card numbers. Data were collected from patients' medication records to record the indications, dose, quantity dispensed, duration of therapy, and coadministered drugs with ceftriaxone; the criteria used for describing the interactions between ceftriaxone and other coadministered drugs were obtained from http://drugs.com website [25]. A systematic random sampling method was used to select the sample records from the sampling frame. Ethical clearance was obtained from the Institutional Review Board (IRB) of Aksum University College of Health Sciences and Comprehensive Specialized Hospital. A permission letter was also obtained from the Tigray Regional Health Bureau with protocol No. Ref 698/7767/16. The personal identifier of the patients was omitted during the medical record review to maintain confidentiality. This paper was assessed for quality presentation using the Strengthening the Report of Observational Studies in Epidemiology (STROBE) guideline (File S1).

### 2.7. Data Analysis

The data were checked for completeness and entered into Statistical Package for Social Sciences (SPSS) version 20, which was used to analyze the data. Based on WHO criteria, indication, dose, frequency, and duration were used as major measurements against the STG of Ethiopia. The appropriateness of ceftriaxone therapy was evaluated based on these four parameters (criteria in the guideline), in which ceftriaxone use was termed appropriate when all four parameters were fulfilled and inappropriate when any one of the four parameters was not fulfilled. A descriptive analysis was employed, and the results are presented in tables and a figure.

## 3. Results

A total of 800 patients' medication records from both hospitals (400 each) were assessed in this study. In Kahsay Abera Hospital, the sex proportion of patients was 271 (67.8%) male and 129 (32.2%) female. The age distribution of the patients included in the study includes 87 (21.8%) infants and children and 313 (78.2%) adults. Out of the 400 patients in Mearg Hospital, 227 (56.8%) were male, 173 (43.2%) were female, 117 (29.2%) were infants and children, and the rest, 283 (70.8%), were adults. The majority of treatment durations were in the range of 2-7 days in Kahsay Abera and Mearg hospitals, accounting for 69% and 88%, respectively. In Kahsay Abera and Mearg hospitals, 341 (85.2%) and 370 (92.5%) patients have no ceftriaxone drug interaction with coadministered drugs, respectively. All patients were not hypersensitive to ceftriaxone in both hospitals. As described in Table 1, the empiric use of ceftriaxone in Mearg Hospital (93.8%) is higher than in Kahsay Abera Hospital (65.5%). But in both hospitals, the reason for using ceftriaxone for prophylaxis purposes is low (23.5% vs. 2%). The results indicate that the majority of patients were given a 2 gm ceftriaxone daily dose in both Kahsay Abera and Mearg hospitals, 285 (71.25%) and 318 (79.5%), respectively (Table 1). The 4 gm daily dose of ceftriaxone was twofold higher in Mearg Hospital than in Kahsay Abera (4.3% vs. 1.8%).

Among the drugs coadministered with ceftriaxone, metronidazole was proportionally the most frequently coprescribed drug in both Kahsay Abera and Mearg hospitals. Comparatively, paracetamol was more commonly prescribed in Mearg Hospital (11.28%) than in Kahsay Abera (2.99%). In Kahsay Abera Hospital, tramadol (11.28%) was more coadministered than paracetamol (Table 2).

In this study, the top three diseases, indicated for ceftriaxone in both hospitals, were typhoid fever (*n* = 87, for Kahsay Abera; *n* = 132, for Mearg), urinary tract infection (UTI) (*n* = 83, for Kahsay Abera; *n* = 86, Mearg), and pneumonia (*n* = 60, for Kahsay Abera; *n* = 52, for Mearg) in descending order. It was also prescribed for surgical prophylaxis, bacillary dysentery, meningitis, and other diseases in both hospitals (Table 3).

Among the four parameters, the majority of inappropriate use of ceftriaxone was noted in indication errors at both Kahsay Abera (71.2%) and Mearg hospitals (52%). As per the findings, the frequency of administration was almost appropriate in both hospitals. The percentages of inappropriate frequency of administration of ceftriaxone were low in Kahsay Abera (0.7%) and Mearg (2.7%) hospitals (Table 4).

**Figure 1:**
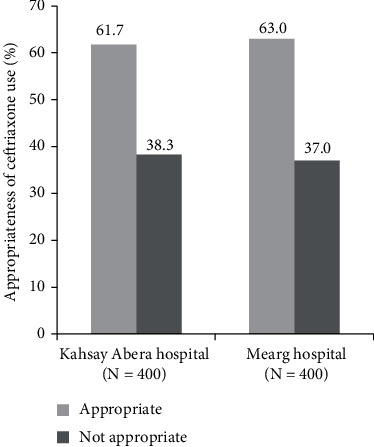
Appropriateness of ceftriaxone therapy in Western zone Tigray hospitals, Ethiopia.

About one-third of patients' charts were not found to comply with the overall evaluation of indication, dose, frequency, and duration of ceftriaxone treatment according to the 2014 STG of Ethiopia for general hospitals. Ceftriaxone therapy in Kahsay Abera (38.3%) and Mearg hospitals (37%) was not appropriate as per the guidelines (Figure 1).

## 4. Discussion

The present study was aimed at evaluating the appropriateness of ceftriaxone use in Western zone Tigray hospitals. The appropriateness of ceftriaxone use in Kahsay Abera and Mearg hospitals was 61.8% and 63%, respectively; these values were higher than some studies performed in other parts of Ethiopia including Dessie Referral Hospital (54.0%) [26], Tikur Anbessa Hospital (12.1%) [27], Ayder Referral Hospital (35.8%) [28], and Gondar University Referral Hospital (19.8%) [29]. The variations may be due to the fact that patient flow is higher in referral hospitals (delayed lab result) than in general hospitals; as a result, many senior physicians usually prescribe ceftriaxone without requesting lab tests, and also, some physicians thought that lab results came out from the less equipped lab setup were not reliable. For instance, in Tikur Anbessa Hospital (the largest hospital in Ethiopia), ceftriaxone was prescribed empirically for most of the cases (87.3%) [27], wherein senior physicians usually prescribe ceftriaxone confidently by experience (clinical judgment), but in general hospitals (in the present study), the patient load is lower and junior prescribers could have less confidence to order ceftriaxone frequently.

Our findings were a little bit lower than the result obtained from a prospective multicenter study of Korea (65.5%) [30]. The difference could be due to the fact that the study carried out in Korea was prospective; evaluation of a patient's drug therapy before dispensing a drug (considering patient outcome) can be the drawback of a retrospective study; moreover, additional criteria (culture and sensitivity) were used in Korea [30].

However, there is still a significant amount of inappropriate use observed in the current study. A high proportion of inappropriate use of ceftriaxone was seen in Kahsay Abera (38.3%) and Mearg (37%) hospitals which were measured based on WHO drug use evaluation criteria against the STG of Ethiopia [19, 22]. Both Kahsay Abera and Mearg hospitals have almost similar appropriateness for ceftriaxone therapy. The possible reason could be that both hospitals are general hospitals, and there could be similar resources (prescribers and diagnostic lab setup). The most frequent cause of inappropriate ceftriaxone use in both Kahsay Abera (71.2%) and Mearg hospitals (52.0%) was an inappropriate indication. Our findings were much higher than the results at Dessie Referral Hospital (6.2%) [26] and Felege Hiwot Hospital (4.7%) [31], and it indicates that there is weakness in both general hospitals with respect to complying with the national STG in selecting the appropriate drugs and also poor diagnostic facilities, whereas the inappropriate use due to wrong duration obtained in this study, Kahisay Abera (20.92%) and Mearg hospitals (29.1%), is less than the result obtained from Dessie Referral Hospital (47.3%) and Felege Hiwot Hospital (59.2%) indicating better performance in the current study but still less than the expected threshold (10%) which is an indicator of another area requiring intervention [26, 31].

In both hospitals understudy, frequency was the least common cause of inappropriate use of ceftriaxone which was less than studies done in other parts of Ethiopia, including Ras-Desta Memorial General Hospital (4.2%) [32], Dessie Referral Hospital (24%) [26], and Felege Hiwot Hospital (13.4%) [31]. The frequency of inappropriate ceftriaxone use as a result of the wrong frequency in this study is also less than the expected threshold (5%) which is one area of strength that should be maintained in both Kahsay Abera and Mearg hospitals.

The top three diseases for which ceftriaxone was prescribed inappropriately at Kahsay Abera and Mearg hospitals were typhoid fever, UTI, and pneumonia. Similar findings were reported in Ayder [28]. But, in Addis Ababa, Zewditu, and Hayat hospitals, pneumonia, meningitis, and sepsis were reported as the most common indications for ceftriaxone, where there is a similarity with pneumonia [33]; one study in Eritrea also indicated that the top two indications for ceftriaxone were pneumonia and sepsis [8]. This indicates that pneumonia and other diseases are prevalent and known to consume the drug in most Ethiopian and Eritrean hospitals. Therefore, continuous training should be provided for the prescribers in both hospitals to improve their appropriate ceftriaxone prescribing habit for the leading sources of errors.

In the present study, among the medications coadministered with ceftriaxone, the top three coprescribed drugs with ceftriaxone in Kahsay Abera Hospital were metronidazole (17.25%), tramadol 68 (11.28%), and diclofenac (8.96%); but in Mearg Hospital, next to metronidazole, drugs like paracetamol and doxycycline were the most common coprescribed medicines along with ceftriaxone. Similar findings were reported in Gondar [29], Addis Ababa [33], Ayder, and Mekelle [28]. The overall pattern in both hospitals indicated that infectious diseases were the major comorbidities. Although there was no major drug interaction in the current study, efforts need to be implemented to reduce the incidence of moderate and major interactions through the drug information center (DIC) at both hospitals to help prescribers select the appropriate drugs (those with minor or no drug interaction) to be used with ceftriaxone [34]. The mean duration of ceftriaxone use for this study in Kahsay Abera was found to be 5.26 days (SD = 2.99), ranging between 0.8% for >14 days and 69.82% for 2-7 days; this study was less than that of Ayder Referral Hospital (7.2 days), Tikur Anbessa Hospital (7.2 days), Felege Hiwot Hospital (5.6 days), and the Korean multicenter study (10.2 days) [26, 31]. The contributing factors for the variations in the mean duration of ceftriaxone use between the Kahsay Abera and other studies could possibly be the variation in the reason for which ceftriaxone is used, i.e., more frequent use of ceftriaxone for conditions requiring a short duration such as prophylaxis and use of inappropriate frequency.

Regarding the dose distribution of ceftriaxone, a 2 gm daily dose was mostly used in both Kahsay Abera (71.25%) and Mearg hospitals (79.5%). The use of a 2 gm daily dose was a bit higher in Mearg Hospital than in Kahsay Abera Hospital; the variation may be due to variations in the indication and frequency of the ceftriaxone therapy in the two hospitals. Our result is almost similar to a study done in Dessie [26], but a study in Tikur Anbessa Hospital indicates the most common prescribed dose of ceftriaxone was 1 gm daily (87.9%) [27]. Comparatively, the use of ceftriaxone for prophylaxis is four times higher in Kahsay Abera Hospital (8%) than in Mearg Hospital (2%). The possible reason could be the surgical service difference in Kahsay Abera and Mearg hospitals. The results of the current study indicate that there are some areas that require intervention to further improve the drug use pattern in Kahsay Abera and Mearg hospitals. One of the interventions is training all the medical staff to have enough awareness of the burden of antimicrobial drug resistance, the risk of drug interactions, and their role as health professionals in combating it. Providing the national updated manuals, including those for STG hospitals, good dispensing, and good prescribing manuals by the regulatory authority will help to improve the medical record system, encourage appropriate use of drugs, and promote rational drug use.

## 5. Limitations of the Study

As the study design was a retrospective cross-sectional study, it confers limitations inherent to this type of design which may underestimate the rate of inappropriate use of ceftriaxone. The evaluation was relied merely on the patients' medical records for which practices might have actually been different on the ground.

## 6. Conclusion

The appropriateness of ceftriaxone use in Kahsay Abera and Mearg hospitals was 61.8% and 63%, respectively, in which about one-third of patients' charts were not compliant with the standard STG treatment guidelines of Ethiopia for general hospitals, and the appropriateness in both hospitals was almost similar. In both hospitals, ceftriaxone was used for empiric purposes. This gives rise to the emergence of resistant pathogens which brings to treatment failure. So, Kahsay Abera and Mearg hospitals need to improve the appropriate use of the right indication for the correct duration of time with the least possible drug interactions. Ceftriaxone should be prescribed according to the national STG, and other analytical studies are required.

## Figures and Tables

**Table 1:** Clinical data of patients with ceftriaxone prescription in hospitals of Western zone Tigray, Ethiopia.

	Kahsay Abera Hospital (*N* = 400)	Mearg Hospital (*N* = 400)	Total (*N* = 800)
	Frequency (%)	Frequency (%)	Frequency (%)
Duration of treatment			
Stat	7 (1.8)	4 (1.0)	11 (1.4)
1 day	82 (20.5)	6 (1.5)	88 (11.0)
2-7 days	279 (69.8)	352 (88.0)	631 (78.9)
8-14 days	29 (7.2)	35 (8.8)	64 (8.0)
>14 days	3 (0.8)	3 (0.8)	6 (8.0)
Total	400 (100)	400 (100.0)	800 (100.0)
Drug interaction			
No interaction	341 (85.2)	370 (92.5)	711 (88.9)
Minor	36 (9.0)	3 (0.8)	39 (4.9)
Moderate	23 (5.8)	24 (6.0)	47 (5.9)
Major	0 (0.0)	3 (0.8)	3 (0.4)
Hypersensitivity			
Not present	400 (100)	400 (100.0)	800 (100.0)
Present	0 (0.0)	0 (0.0)	0 (0.0)
Past medical history			
Not present	268 (67.0)	234 (58.5)	502 (62.8)
Present	132 (33.0)	166 (41.5)	298 (37.3)
Reason for use			
Empiric	262 (65.5)	375 (93.8)	637 (79.6)
Prophylaxis	94 (23.5)	8.0 (2.0)	102 (12.8)
Kinetic (supportive)	44 (11.0)	17 (4.2)	61 (7.6)
Daily dose of ceftriaxone (gram)			
<2	108 (27.0)	65 (16.25)	173 (21.6)
2	285 (71.2)	318 (79.5)	603 (75.4)
4	7 (1.8)	17 (4.8)	24 (3.0)

Key: % = percentage; *N* = number (patients' medical cards).

**Table 2:** Frequently coadministered drugs with Ceftriaxone, in Western zone Tigray hospitals, Ethiopia.

Drug	Frequency	Percent
Kahsay Abera Hospital		
Metronidazole	104	17.25
Tramadol	68	11.28
Diclofenac	54	8.96
Artesunate	39	6.47
Cimetidine	34	5.64
Ferrous sulphate	22	3.65
Antituberculosis drugs	20	3.32
Furosemide	20	3.32
Amoxicillin	19	3.15
Paracetamol	18	2.99
Doxycycline	14	2.32
Spironolactone	12	1.99
Cloxacillin	10	1.66
Others (52 types)	169	28.03
Mearg Hospital		
Metronidazole	39	13.68
Paracetamol	34	11.93
Doxycycline	22	7.72
Cloxacillin	17	5.96
Artesunate	16	5.61
Gentamycin	14	4.91
Coartem	13	4.56
Cimetidine	10	3.51
Albendazole	10	3.51
Others	110	38.60
-	—	—
-	—	—
-	—	—
-	—	—

**Table 3:** The most common indications for ceftriaxone and compliance to respective guidelines in Western zone Tigray hospitals, Ethiopia.

Assessment	Kahsay Abera Hospital (*N* = 400)		Mearg Hospital (*N* = 400)		Total (*N* = 800)	
	*N*	INAP frequency (%)	*N*	INAP frequency (%)	*N*	INAP frequency (%)
Typhoid fever	87	42 (48.3)	132	56 (42.4)	219	98 (44.7)
UTI	83	36 (43.4)	86	21 (24.4)	169	57 (33.7)
Pneumonia	60	30 (50.0)	52	22 (42.3)	112	52 (46.4)
Surgical prophylaxis	46	4 (8.7)	41	8 (19.5)	87	12 (13.8)
Bacillary dysentery	31	13 (41.9)	26	14 (53.9)	57	27 (47.4)
Meningitis	19	10 (52.6)	21	6 (28.6)	40	16 (40.0)
Gonorrhea	10	4 (40.0)	-	-	10	4 (40.0)
Others	64	14 (21.9)	49	21 (42.9)	113	35 (30.0)
Total	400	153 (38.3)	400	148 (37.0)	800	301 (37.6)

Key: *N*: total number of assessed patients' medical cards; %: percentage; INAP: inappropriate (not comply with the guideline); -: not found.

**Table 4:** Distribution of inappropriate use of ceftriaxone among the four indicators in Western zone Tigray hospitals, Ethiopia.

Parameters	Kahsay Abera Hospital	Mearg Hospital	Total
	No. of errors (%)	No. of errors (%)	No. of errors (%)
Indication	109 (71.2)	77 (52.0)	186 (61.8)
Dose	11 (7.2)	24 (16.2)	35 (11.6)
Frequency	1 (0.7)	4 (2.7)	5 (1.7)
Duration	32 (20.9)	43 (29.1)	75 (24.9)
Total	153 (100.0)	148 (100.0)	301 (100.0)

Key: No. of errors: number of errors; %: percentage.

## Data Availability

All the relevant data are included within the manuscript, but the datasets used during the current study are available from the corresponding author on reasonable request.

## Abbreviations

DIC: Drug Information Center
DUE: Drug use evaluation
IRB: Institutional Review Board
PCM: Paracetamol
SPSS: Statistical Package for Social Sciences
STG: Standard treatment guidelines
UTI: Urinary tract infections.

## Supplementary Materials

Supplementary Materials S1 file: Strengthening the Report of Observational Studies in Epidemiology (STROBE) guideline.
