# Supplementary material for: Ceftriaxone Use Evaluation in Western Zone Tigray Hospitals, Ethiopia: A Retrospective Cross-Sectional Study
Source: Biomed Res Int. 2023 Nov 13;2023:7688896. doi: 10.1155/2023/7688896 (PMC10661873; doi:10.1155/2023/7688896)
Supplement: Supplementary Materials — S1 file: Strengthening the Report of Observational Studies in Epidemiology (STROBE) guideline. [file 7688896.f1.pdf]

STROBE Statement—Checklist of items that should be included in reports of *cross-sectional studies*

Ceftriaxone Use Evaluation in Western Zone Tigray Hospitals, Ethiopia: a retrospective cross-sectional study

Tewodros Shegute1, Mulugeta Hiruy2, Haftom Hadush3, Leake Gebremeskel1*

1 Pharmacology and Toxicology Unit, Department of Pharmacy, College of Health Sciences, Aksum University, Aksum, Ethiopia

2Department of Biomedical Sciences, College of Health Sciences, Aksum University, Aksum, Ethiopia

3Department of Medical Laboratory Technology, College of Health Sciences, Aksum University, Aksum, Ethiopia

	Item No	Recommendation
**Title and abstract**	1	Indicate the study's design with a commonly used term in the title or the abstract<br>*-Retrospective cross-sectional study design is indicated and included in the title and abstract*
		(*b*) Provide in the abstract an informative and balanced summary of what was done and what was found<br>*-Informative abstract is provided in the manuscript from line 25-5, page 2*
**Introduction**		
Background/rationale	2	Explain background and rationale for the investigation being reported<br>*-From page 2-4,the background/rational is explained*
Objectives	3	State specific objectives, including any pre-specified hypotheses<br>*-The aim of the study is described in the introduction, last paragraph, page 4,line 84-86*
**Methods**		
Study design	4	Present key elements of study design early in the paper<br>*-Key elements of study design are included in the method part of the manuscript*
Setting	5	Describe the setting, locations, and relevant dates, including periods of recruitment, exposure, follow-up, and data collection<br>*-The setting and location are well described in the method section, page 4 ; and data collection, the period are described in page 5 and 6*
Participants	6	Give the eligibility criteria, and the sources and methods of selection of participants<br>*-This included in the manuscript, page 5, line 102-106*
Variables	7	Clearly define all outcomes, exposures, predictors, potential confounders, and effect modifiers. Give diagnostic criteria, if applicable<br>*-Not applicable*
Data sources/ measurement	8*	For each variable of interest, give sources of data and details of methods of assessment (measurement). Describe comparability of assessment methods if there is more than one group<br>*-Not applicable*
Bias	9	Describe any efforts to address potential sources of bias<br>-As we use retrospective cross-sectional study design to review records of patients, potential bias was less, to select the card we used systematic random sampling to avoid bias
Study size	10	Explain how the study size was arrived at<br>- Sample size was determined using single population proportion formula with a proportion (p=0.5) of inappropriate ceftriaxone utilization and confidence level of 95% with a z-value of 1.96 (P=0.05) with 10% contingency; and systematic random

		sampling was employed. The first patients' medical chart was selected using simple random sampling technique then every 3rd chart was selected until the desired sample size was reached; 23 medical cards (in each hospital) were incomplete and were excluded; hence only 400 cards were analysed in each hospital
Quantitative variables	11	Explain how quantitative variables were handled in the analyses. If applicable, describe which groupings were chosen and why<br>*-Not applicable*
Statistical methods	12	Describe all statistical methods, including those used to control for confounding<br>-The study is descriptive and the data were checked for completeness and entered in SPSS
		(*b*) Describe any methods used to examine subgroups and interactions<br>*-Not applicable*
		(*c*) Explain how missing data were addressed<br>-Incomplete cards were excluded, 10% contingency was used and we got 400 complete cards from each hospital
		(*d*) If applicable, describe analytical methods taking account of sampling strategy<br>-Not applicable
		(*e*) Describe any sensitivity analyses<br>*-Not applicable*
**Results**		
Participants	13*	Report numbers of individuals at each stage of study—eg numbers potentially eligible, examined for eligibility, confirmed eligible, included in the study, completing follow-up, and analysed<br>*-Not applicable*
		(b) Give reasons for non-participation at each stage<br>*-Not applicable*
		(c) Consider use of a flow diagram<br>*-Not applicable*
Descriptive data	14*	Give characteristics of study participants (eg demographic, clinical, social) and information on exposures and potential confounders<br>-Characteristics of patients is described from line 146-151, page 7
		(b) Indicate number of participants with missing data for each variable of interest<br>*-Not applicable*
Outcome data	15*	Report numbers of outcome events or summary measures<br>*-Not applicable*
Main results	16	Give unadjusted estimates and, if applicable, confounder-adjusted estimates and their precision (eg, 95% confidence interval). Make clear which confounders were adjusted for and why they were included<br>--Results are reported using percentage with 95% confidence and frequency; with tables and figures in the result section
		(*b*) Report category boundaries when continuous variables were categorized<br>-Not applicable
		(*c*) If relevant, consider translating estimates of relative risk into absolute risk for a meaningful time period<br>*-Not applicable*
Other analyses	17	Report other analyses done—eg analyses of subgroups and interactions, and sensitivity analyses

		-Not applicable
**Discussion**		
Key results	18	Summarise key results with reference to study objectives<br>-Key findings are summarized in the discussion part of the manuscript in the first paragraph, page 12
Limitations	19	Discuss limitations of the study, taking into account sources of potential bias or imprecision. Discuss both direction and magnitude of any potential bias<br>*-The limitation of the study is well described in page 15, line 288-291*
Interpretation	20	Give a cautious overall interpretation of results considering objectives, limitations, multiplicity of analyses, results from similar studies, and other relevant evidence<br>*-It is included in the discussion section of the paper*
Generalisability	21	Discuss the generalisability (external validity) of the study results<br>*-The generalizability of the finding is described the conclusion, page 16, line 292-301*
**Other information**		
Funding	22	Give the source of funding and the role of the funders for the present study and, if applicable, for the original study on which the present article is based<br>*-There was no any fund for this study*

*Give information separately for exposed and unexposed groups.

Note: An Explanation and Elaboration article discusses each checklist item and gives methodological background and published examples of transparent reporting. The STROBE checklist is best used in conjunction with this article (freely available on the Web sites of PLoS Medicine at http://www.plosmedicine.org/, Annals of Internal Medicine at http://www.annals.org/, and Epidemiology at http://www.epidem.com/). Information on the STROBE Initiative is available at www.strobe-statement.org.
